# COLD-PCR Amplification of Bisulfite-Converted DNA Allows the Enrichment and Sequencing of Rare Un-Methylated Genomic Regions

**DOI:** 10.1371/journal.pone.0094103

**Published:** 2014-04-11

**Authors:** Elena Castellanos-Rizaldos, Coren A. Milbury, Elli Karatza, Clark C. Chen, G. Mike Makrigiorgos, Anne Merewood

**Affiliations:** 1 Division of DNA Repair and Genome Stability, Department of Radiation Oncology, Dana-Farber Cancer Institute, Harvard Medical School, Boston, Massachusetts, United States of America; 2 Division of Neurosurgery, University of California San Diego, San Diego, California, United States of America; 3 Division of Medical Physics and Biophysics, Department of Radiation Oncology, Dana-Farber Cancer Institute, Brigham and Women's Hospital, Harvard Medical School, Boston, Massachusetts, United States of America; 4 Division of General Pediatrics, Boston Medical Center and Boston University, Boston, Massachusetts, United States of America; City of Hope, United States of America

## Abstract

Aberrant hypo-methylation of DNA is evident in a range of human diseases including cancer and diabetes. Development of sensitive assays capable of detecting traces of un-methylated DNA within methylated samples can be useful in several situations. Here we describe a new approach, *fast*-COLD-MS-PCR, which amplifies preferentially un-methylated DNA sequences. By employing an appropriate denaturation temperature during PCR of bi-sulfite converted DNA, *fast*-COLD-MS-PCR enriches un-methylated DNA and enables differential melting analysis or bisulfite sequencing. Using methylation on the MGMT gene promoter as a model, it is shown that serial dilutions of controlled methylation samples lead to the reliable sequencing of un-methylated sequences down to 0.05% un-methylated-to-methylated DNA. Screening of clinical glioma tumor and infant blood samples demonstrated that the degree of enrichment of un-methylated over methylated DNA can be modulated by the choice of denaturation temperature, providing a convenient method for analysis of partially methylated DNA or for revealing and sequencing traces of un-methylated DNA. *Fast*-COLD-MS-PCR can be useful for the detection of loss of methylation/imprinting in cancer, diabetes or diet-related methylation changes.

## Introduction

The role of DNA promoter methylation in cancer genetics has been widely studied and some of its mechanisms elucidated, however, the implication of DNA hypomethylation in oncogenesis is less well understood. Studies show a decrease in 5-methylcytosine levels in cancer tissues compared to their surrounding normal tissue in colon adenocarcinomas, Wilms' tumors and ovarian epithelial carcinomas, among others. The relationship of DNA hypomethylation to tumorigenesis is important to consider in view of cancer therapies that operate by decreasing DNA methylation. [1]. Furthermore, during gonad development gene imprinting takes place through parent of origin allele-specific methylation processes. The loss of imprinting (i.e. un-methylation of a previously methylated allele) may result in placental defects as well as in a series of genetic conditions that could increase the risk of cancer [2], [3]. Loss of methylation is also important in diabetes, where differential methylation occurs in genes that encode pre-proinsulin where β-cells are mainly un-methylated compared to other cell types. Detection of un-methylated pre-proinsulin DNA in serum has been associated to pancreatic β-cell death. Thus detecting DNA un-methylation at early stages of disease progression may provide actionable interventions in pre-diabetic stages [4]. Given such possibilities, the identification of small traces of un-methylated DNA within methylated DNA is of significance and is clinically desirable in cancer, diabetes and in other fields where epigenetics provides a potential link between lifestyle (diet, alcohol, smoking) with diabetes, obesity and cancer [5], [6].

A range of methods currently exist for detecting methylated or un-methylated DNA including methylation-specific PCR [7]–[10], differential enzymatic restriction methods [11], isothermal amplification methods [12], high resolution melting [13] or bisulfite sequencing [14] carried out as traditional Sanger sequencing or using next generation sequencing technologies. By revealing the status of individual CpG di-nucleotides, sequencing provides the most complete information on the methylation status of a target region. On the other hand real-time methylation-specific PCR is able to detect the presence of rare un-methylated alleles among an excess of methylated alleles or vice versa, something which is difficult to achieve with bisulfite sequencing alone. Below we demonstrate that Co-amplification at Lower Denaturation temperature PCR, COLD-PCR, [15]–[17], can be adapted for detection and sequencing of traces of un-methylated alleles within a population of methylated alleles. This approach relies on the substitution of the denaturation temperature (typically 95–98°C) by a decreased denaturation temperature (critical denaturation temperature, T_c_). This allows the preferential amplification of sequences that have a lower melting temperature. Detection can be performed using an intercalating dye and melting analysis, followed by direct sequencing of the resulting amplicons without the need for specific reagents or use of special instrumentation.

We chose methylation of the gene encoding for O6 methylguanine methyl transferase (MGMT) as an example to validate COLD Methylation-specific (MS)-PCR in DNA from glioblastoma or infant blood samples. MGMT is of particular importance in glioblastoma. It encodes an evolutionarily conserved protein whose primary function is to repair guanine nucleotides that are alkylated at the O6 position [18]. As O6-methyl guanine constitutes the major cytotoxic lesion related to temozolomide, the only efficacious chemotherapy against glioblastoma, MGMT serves as the primary determinant of therapeutic response. MGMT promoter methylation leads to decreased expression of MGMT, and this methylation pattern have been associated with improved therapeutic response by three independent, well-conducted clinical trials, including the EORTIC-NCIC, NOA-8, and the Nordic trials [19]. As such, methodologies for sensitive and reproducible detection MGMT promoter methylation would better facilitate clinical management of glioblastoma patients. Using COLD-MS-PCR we demonstrate that we are able to amplify and sequence 0.05% of un-methylated MGMT DNA within an excess of 99.95% of methylated MGMT DNA.

## Materials and Methods

### DNA, clinical samples and bisulfite treatment

Un-methylated and enzymatically methylated DNA (Millipore Corporation, Billerica, MA, USA) were obtained and subjected to bisulfite treatment using the EpiTect Bisulfite kit following manufacturer's recommendations (Qiagen Inc.). Additional Epitect bisulfite-converted un-methylated and methylated controls were obtained from Qiagen. Serial dilutions of un-methylated and methylated DNA were prepared.

Two sets of clinical samples were tested, chosen for their potential to harbor methylation (or lack of methylation) on the *MGMT* gene. Glioma tumor tissue specimens (N = 20), obtained from the Division of Neurosurgery at the Beth Israel – Deaconess Medical Center; and blood samples (N = 10) isolated from blood of infants at the Department of Pediatrics, Boston Medical Center. Infant samples were obtained after written informed consent by the mothers and following approval by the IRB committee of Boston Medical Center. Glioma samples were also obtained following written consent prior to surgery (BIDMC Division of Neurosurgery) and following approval by the IRB committee of DFCI/BIDMC. These samples were processed for DNA isolation using the DNAeasy Blood & Tissue Kit (Qiagen) as described [20], followed by bisulfite sequencing.

### Methylation-specific *fast*-COLD-PCR (*fast*-COLD-MS-PCR)

*Fast*-COLD-PCR is the simplest version of COLD-PCR [15]. It leads to the preferential amplification of sequences containing genomic alterations that result to a reduction of the melting temperature of the PCR amplicon and combines well with downstream detection formats including high resolution melting [21]–[24]. This property is exploited here for sensitive detection of un-methylated DNA. During sodium bisulfite conversion of DNA, deamination of un-methylated cytosines generates uracils whereas 5-methylcytosines remain unaffected. In a subsequent PCR reaction uracil residues are amplified as thymines and 5-methyl-cytosines as cytosines [14]. This results in a large difference in GC content and melting temperature differential changes that can then be exploited by COLD-PCR for selectively amplifying un-methylated DNA.

PCR reactions, specific for bisulfite-converted DNA, were designed and optimized to amplify a region of *MGMT* (O-6-methyl-guanine-DNA methyltransferase) gene. A pre-amplification of 379 bp using primers that bind target regions without CpG sites 5′-TTTGTTTTTTTTAGGTTTT-3′ and 5′-CCAAAAACCCCAAACCCGA-3′ with an annealing temperature of 48°C was performed as reported [25]. A second, nested PCR reaction was designed using 5′-GGATATGTTGGGATAGTT-3′ [25] and 5′-CACCTAAAAAACACTTAAAAC-3′ with an annealing temperature of 52°C to produce a 255 bp amplicon. The second reaction was performed in a *fast*-COLD-PCR format to favor the amplification of un-methylated DNA. Alternatively a nested conventional PCR reaction was performed for comparison. PCR reagents for both first and nested PCR reactions comprised 1× Colorless Gotaq reaction buffer, 2 mM MgCl_2_, 0.2 mmol/L each dNTP, 0.25 µM each primer, 0.1× LCGreen+ dye (Idaho Technologies Inc.), and 1 unit/ul GoTaq polymerase (Promega, Madison, WI, USA). *Fast*-COLD-MS-PCR thermocycling protocol for amplification on the SmartCycler II (Cepheid, Inc.) was (5 cycles of conventional PCR followed by 35 cycles at the selected critical denaturation temperature (T_c_) for 10 s, 52°C for 30 s and 72°C for 20 s with a final extension at 72°C). Conventional PCR protocol was (initial denaturation at 95°C for 2 min, then 40 cycles at 95°C for 30 s, 52°C for 30 s and 72°C for 20 s with a final extension at 72°C for 5 min). Immediately following the extension step of the nested reaction, PCR amplicons were subjected to melting curve analysis on the Smartcycler II. Amplicons were processed for Sanger sequencing. Experiments were repeated three independent times.

## Results and Discussion

**Figure 1:**
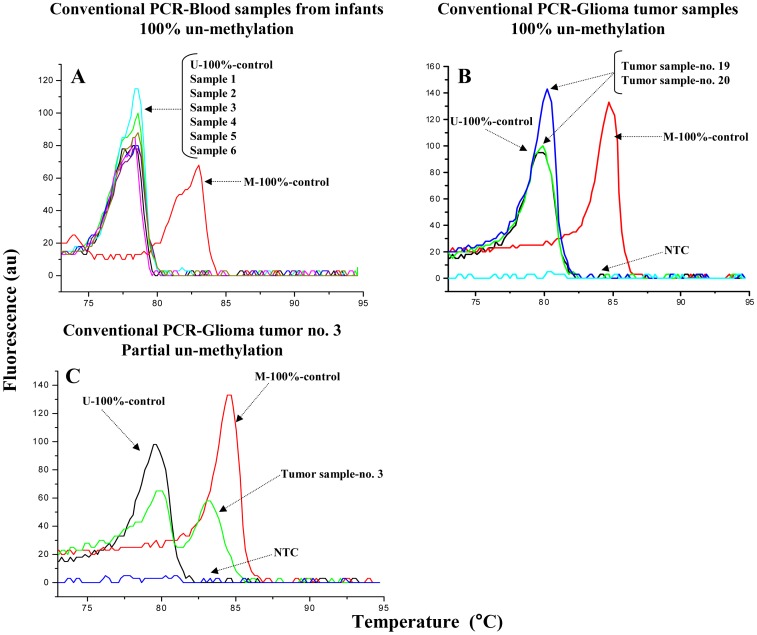
Melting profiles of bisulfite-converted DNA from clinical samples following conventional PCR. Post-PCR melting profiles of the 255 bp bisulfite-converted *MGMT* gene amplicon after conventional PCR. Examples of fully un-methylated DNA samples isolated from infant blood (**Panel A**) and glioma samples (**Panel B**) are depicted. 100% methylated (M) and 100% un-methylated (U) DNA controls are used as reference standards, demonstrating a ∼5°C melting temperature difference among the two. **Panel C.** A glioma sample with mixed methylation/unmethylation pattern is shown.

Melting profiles of *MGMT* target gene regions using conventional PCR-amplification of bisulfite-converted DNA from glioma tumor samples and infant blood samples, along with fully un-methylated and fully methylated DNA controls are depicted on **Figure 1, A-C**. All the samples from infant blood generated amplicons with melting profiles matching the 100% un-methylated control DNA at the *MGMT* promoter region, and six representative samples are depicted on **Figure 1A**. The fully un-methylated and fully methylated DNA produced amplicons with melting temperatures (T_m_) that differed by approximately 5°C (**Figure 1A**). Out of 20 glioma samples tested, 19 samples generated amplicons with melting profiles consistent with 100% un-methylated control DNA. **Figure 1B** depicts two such un-methylated glioma samples. A single glioma sample (no. 3) generated a melting profile consistent with a mix of methylated plus un-methylated DNA sequences at the *MGMT* promoter region, which was different from the other glioma tumor samples used to explain the reliability and stability of the approach (**Figure 1C**).

**Figure 2:**
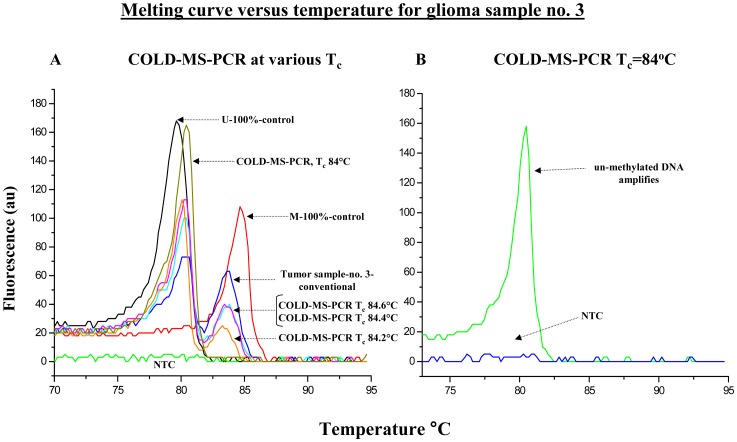
Melting profiles of bisulfite-converted DNA from clinical samples following *fast*-COLD-MS-PCR. The effect of lowering the denaturation temperature in PCR is depicted. **Panel A.** Glioma sample no. 3 was subjected to different critical denaturation temperature-T_c_ during *fast*-COLD-MS-PCR. The modulation of the preferential amplification of the un-methylated DNA fraction is shown. **Panel B.** *fast*-COLD-MS-PCR performed at a T_c_ of 84°C demonstrates that the amplification of the methylated DNA fraction is completely inhibited.

Next we demonstrated that by modulating the denaturation temperature during PCR, we can select with different efficiency the un-methylated DNA fraction of a give sample. In **Figure 2**, **Panel A**, we analyzed the effect of gradually lowering the denaturation temperature during PCR, using for this purpose glioma sample no. 3 with a mixture of un-methylated and methylated DNA. By gradully shifting the T_c_ during PCR, a gradual shifting of the un-methylated and methylated DNA portions is observed. When a T_c_ of 84°C was used during *fast*- COLD-MS-PCR, the methylated peak disappeared in the subsequent melting profiles, consistent with an almost exclusive amplification of un-methylated DNA (**Figure 2B**).

**Figure 3:**
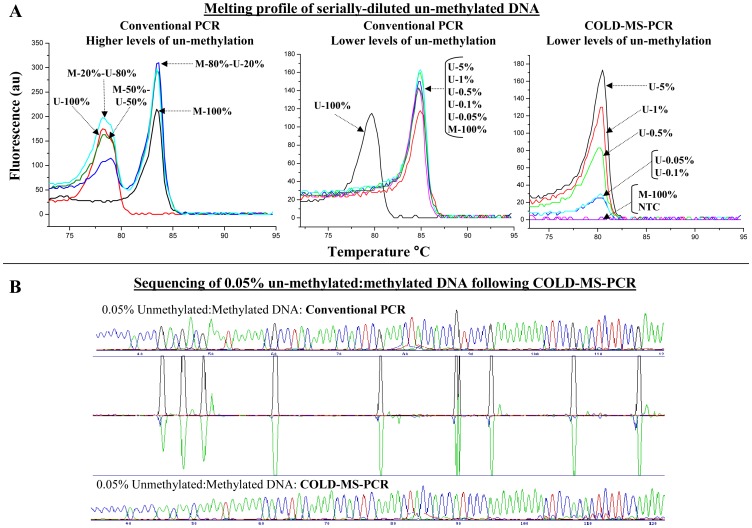
Detection of un-methylated DNA within different abundances of methylated DNA background by conventional or *fast*-COLD-MS-PCR. **Panel A.** Post-PCR melting profile of the 255 bp bisulfite-converted-specific amplicon after conventional PCR or COLD-PCR. Serial dilutions of un-methylated (U) to methylated (M) genomic DNA are depicted (top half). Higher abundances of un-methylated DNA can be discriminated from methylated DNA by the melt peak, whereas lower abundances are only detectable if *fast*-COLD-MS-PCR replaces conventional PCR. **Panel B.** Sanger sequencing results of the 0.05% un-methylated (U): methylated (M) DNA sample as amplified by conventional and *fast*-COLD-MS-PCR are shown (bottom half). Chromatograms are aligned and compared using SeqDoc, and the CpG methylation positions are revealed in the middle panel.

To examine the lowest fraction of un-methylated MGMT DNA within an excess of methylated MGMT DNA that can be preferentially amplified, detected and sequenced using *fast*- COLD-MS-PCR we tested serial dilutions of un-methylated to methylated DNA (**Figure 3A**). Using conventional MS-PCR, fractions of un-methylated-to-methylated DNA of 20–80% generate the anticipated double peaks in their melting profiles. However, in fractions below 5% the methylated peak cannot be observed in either the melting profiles or the sequencing chromatograms. In contrast, *fast*-COLD-MS-PCR can enrich and detect the un-methylated DNA down to 0.05%. Furthermore, direct sequencing of the product from an initial 0.05% un-methylated DNA reveals un-methylated C at individual CpG sequence positions **Figure 3B**.

*Fast*-COLD-MS-PCR utilizes primers that bind sequence positions that do not contain CpG sites, i.e. they are neutral in regards to CpG methylation. This is a potential advantage over approaches that utilize methylation-specific primers, such as MS-PCR or methyl-Light [7], [8], as the methylation status of the primer binding sites ideally should not pre-determine which fraction of the target amplicons can be studied. Approaches like COBRA [11] or methylation sensitive high resolution melting [13] employ CpG-neutral primer binding sites and detect low levels of un-methylation, but they do not enable bisulfite sequencing of the product. The ability of *fast*-COLD-MS-PCR to modulate the enrichment effect using graded denaturation temperatures provides a convenient method for analysis of partially methylated DNA or for revealing and sequencing traces of un-methylated DNA. Using *fast*-COLD-MS-PCR enabled the detection of traces of un-methylated sequences by virtue of the T_m_-lowering effect of C>T transversions. On the other hand using different versions of COLD-PCR (*full*-COLD-PCR [26]; ice-COLD-PCR [27]) it should also be possible to reverse the process, i.e. enrich small amounts of methylated DNA within an excess of un-methylated DNA.

In summary, COLD-PCR technology can be adapted to reveal traces of un-methylated DNA within a high excess of methylated DNA.

When *fast*-COLD-MS-PCR was used to analyze bisulfite-converted clinical samples and serially-diluted un-methylated DNA in methylated DNA, low abundances of un-methylated MGMT DNA, down to 0.05%, could be preferentially enriched and sequenced. Thereby one can enrich and sequence traces of un-methylated DNA which can precede disease progression in cancer and diabetes, can be predictive of chemotherapy-caused remission and are linked to diet and lifestyle-induced epigenetic changes.
